# Association between lifestyle behaviors and body mass index with blood pressure classifications among older adults with hypertension in China

**DOI:** 10.3389/fpubh.2025.1610715

**Published:** 2025-07-03

**Authors:** Jian Wu, Yue Chen, Yudong Miao, Quanman Li, Clifford Silver Tarimo, Nengguang Dai, Qiuping Zhao, Yadong Niu

**Affiliations:** ^1^Department of Health Management, College of Public Health, Zhengzhou University, Zhengzhou, China; ^2^Department of Science and Laboratory Technology, Dar es Salaam Institute of Technology, Dar es Salaam, Tanzania; ^3^Grassroots Health and Health Department, Health Commission of Henan Province, Zhengzhou, China; ^4^Henan Key Laboratory for Health Management of Chronic Diseases, Central China Fuwai Hospital, Central China Fuwai Hospital of Zhengzhou University, Zhengzhou, China

**Keywords:** lifestyle behaviors, body mass index, blood pressure classifications, older adults, hypertension

## Abstract

**Objective:**

This study aimed to examine the association between lifestyle behaviors and body mass index (BMI) along with their potential interactions with the severity of blood pressure (BP) classifications among older adults with hypertension.

**Methods:**

Among 17,441 participants, lifestyle behaviors were assessed, including smoking, drinking, dietary patterns, physical activity, and sleeping. Multinomial logistic regression was used to examine the association between lifestyle behaviors and BMI with BP classifications, and multiplicative interactions were included to estimate potential interactions. To explore variations, analyses were also stratified by BMI.

**Results:**

High-risk dietary pattern and obesity were negatively associated with lower BP classifications, with odds ratios (ORs) and 95% confidence intervals (CIs) for normal BP, high-normal BP, and grade 1 hypertension in high-risk dietary pattern were 0.74 (0.57–0.95), 0.69 (0.54–0.90), 0.75 (0.59–0.95), and the ORs (95% CIs) in obesity were 0.61 (0.53–0.69), 0.77 (0.67–0.89), 0.82 (0.73–0.93). Compared with never drinking, former drinkers had higher odds of having normal BP (OR: 1.45, 95% CI: 1.15–1.82), high-normal BP (OR: 1.31, 95% CI: 1.02–1.67), and grade 1 hypertension (OR: 1.26, 95% CI: 1.01–1.58). The multiplicative interaction between drinking status and BMI was found on BP classifications (*P*_forinteraction_ <0.05), and the effects of former drinking and low PA level on BP control were significant in overweight and underweight groups, respectively (*P* < 0.05).

**Conclusions:**

Poor diet and obesity are associate with severe BP, particularly among non-drinking older adults, suggesting targeted interventions in rural primary care.

## 1 Introduction

Hypertension is a prevalent cardiovascular disease (CVD) and remains the leading cause of morbidity and mortality in CVD events worldwide (1, 2).

Currently, the global prevalence of hypertension is reaching critical levels, mainly driven by the population aging and increased exposure to unhealthy lifestyle risk factors (3, 4). According to the latest report by the World Health Organization, the number of individuals aged 30–79 years diagnosed with hypertension has surged from 650 million in 1990 to 1.28 billion in 2019 (5). Although the overall global prevalence of hypertension is on the rise, significant disparities persist across countries. High-income countries (HICs) have experienced a slight decline in hypertension rates, while low and middle-income countries (LMICs) have significantly risen (4). Compared to HICs, the low levels of awareness, treatment and control rates in LMICs have consequently contributed to large disparities in the regional burden of hypertension (6). Furthermore, given the complex complications associated with hypertension such as stroke, renal failure, myocardial infarction, and heart failure (7), there has been a marked increase in disability adjusted life years and mortality linked to hypertension between 1990 and 2015 (8). Therefore, actively controlling blood pressure (BP) and reducing the incidence of hypertension hold essential public health significance for promoting population health and alleviating the global disease burden.

Previous studies have identified various risk factors for hypertension, including non-modifiable risk factors such as aging, race, ethnicity, family history, and co-existing diseases, and modifiable risk factors such as unhealthy lifestyle behaviors (9). Moreover, growing evidence has supported that these modifiable lifestyle factors, including smoking, drinking, diet, and physical activity (PA) are not only strongly associated with hypertension (10, 11), but also can be considered as first-line interventions for managing the condition (2). As a result, more attention should be paid to the role of healthy lifestyle behaviors in controlling BP levels, which may help lower the risk of CVD-related morbidity and mortality (12, 13).

Additionally, overweight and obesity are closely associated with the increased risk of hypertension. In the United States, three-quarters of people with hypertension were either overweight or obese (14). An epidemiological study found that 65%−75% of primary hypertension cases were attributable to overweight and obesity (15). To our knowledge, the body mass index (BMI) which is acknowledged as a common measure index of obesity (16), has been used in previous studies to assess the impact of obesity on the risk of elevated BP.

Growing evidence has emerged in recent years regarding the interaction effects of particular lifestyle factors and BMI on the risk of hypertension. Such as Sun et al. (17) used data from the China Health and Nutrition Survey (2011–2015) to explore the interaction effect between overweight/obesity (defined by BMI, WC, WHR) and alcohol consumption on hypertension risk. The findings indicated that BMI and WC, appear to interact synergistically with alcohol consumption to increase the risk of hypertension in the Chinese population. Crump et al. (18) used Swedish conscript data (1969–1997) and Poisson regression models to determine the interactive effects of BMI and physical fitness on the risk of hypertension. They found that high BMI and low aerobic capacity in late adolescence were independently associated with higher risk of hypertension in adulthood and had a negative interaction.

In summary, the associations between lifestyle behaviors or BMI with BP have been well-established. While some studies have explored how these factors interact to affect BP, most have focused on individual lifestyle behaviors rather than examining multiple key behaviors together. Furthermore, most previous studies have focused on BP status in the general population, with relatively few examining hypertensive individuals, particularly those aged 65 years and older. To fill the gap, we explored the effect of lifestyle and BMI, along with their potential interactions, on BP classifications in a large cross-sectional survey of older adults with hypertension.

## 2 Materials and methods

### 2.1 Study design, data collection and population

This cross-sectional survey was conducted from 1 July to 31 August 2023, in Jia County, Henan Province, China, using the cluster sampling method. Data collection was carried out by a Healthy Lifestyle Research Team specializing in public health research, consisting of 62 trained professionals (48 field investigators, 12 medical staff and 10 data quality controllers). All personnel completed standardized training in: (1) lifestyle questionnaire; (2) standardized anthropometric measurements (height, weight, waist circumference, and blood pressure). Quality assurance protocols were implemented, including real-time field supervision, immediate data verification during collection, and post-collection questionnaire audits to ensure data integrity and data quality.

All the participants were included in the National Basic Public Health Service Program. After excluding individuals with mental disorders, secondary hypertension, and those unable to complete the questionnaire and physical examination, a total of 18,963 older adults (aged 65 years or older) with hypertension were included in the study. The study was approved by the Life Science Ethics Committee of Zhengzhou University (Registration number: 2023-318), and written informed consent was obtained from all participants.

After applying the exclusion criteria, we excluded participants with missing data on lifestyle behaviors and BMI (*n* = 1,398), as well as those lacking systolic blood pressure (SBP) and diastolic blood pressure (DBP) measurements (*n* = 124). Finally, 17,441 participants were included in the final analysis (Supplementary Figure 1).

### 2.2 Assessment of covariates

Covariate information collected through a structured questionnaire primarily included several demographic characteristics variables. These included age (65–74 years, 75–84 years, ≥85 years), gender (male and female), education levels (primary school or below, middle school, high school or above) (19), annual household income (<10,000 yuan, 10,000–19,999 yuan, 20,000–34,999 yuan, ≥35,000 yuan), and use of antihypertensive medication (yes or no).

### 2.3 Assessment of lifestyle behaviors

Following the approach of previous studies (20, 21), five lifestyle behaviors such as smoking, drinking, diet, PA, and sleep were included in our analysis. Smoking status was assessed using the question “Do you smoke?” and categorized into current, former, or never smoking based on the participants' responses (22). Similarly, drinking status was assessed with the question “Have you consumed alcohol more than 12 times in the past 12 months?” (23) and were categorized into current, former, or never drinking. Dietary status was assessed using a Food Frequency Questionnaires, and five frequency categories including never/rarely, monthly, 1–3 days/week, 4–6 days/week, or daily were provided to choose for participants by their dietary intakes in the last year (24). In this study, we selected three main food items (vegetables, fruits, and red meat) particularly emphasized by the American Heart Association and American College of Cardiology to assess the dietary status (25). Notably, participants who consumed vegetables and fruits daily, along with red meat 1–6 days per week, were classified as following a low-risk dietary pattern (26). Sleep behavior was assessed by asking participants to report their average nightly sleep duration, excluding time spent in bed while not asleep. The low-risk group was defined as those who reported getting 7–9 h of sleep per day (27). PA status was assessed by asking participants about their types, frequency, and duration of PA in the previous week. Following the International Physical Activity Questionnaire, the metabolic equivalent of task (MET) was calculated to determine total PA in MET-minutes per week. These calculations categorized PA levels into three groups: low, moderate, and high (28).

To capture the overall lifestyle health status of participants, each lifestyle behavior was assigned a score reflecting its health level, with higher scores indicating healthier behaviors (29). Smoking status was scored on a three-point scale: 1 point for current smokers, 2 points for former smokers, and 3 points for never smokers. Similarly, drinking status was assigned 1–3 points representing current, former, and never, respectively. Dietary patterns were provided 1–2 points from the high-risk group to the low-risk group while sleeping status was scored based on the level of risk (high-risk = 1 point, low-risk = 2 points). Finally, we also allocated PA with 1–3 points following the PA levels from lowest to highest.

### 2.4 Assessment of body mass index

BMI was calculated as weight in kilograms divided by squared height in meters (kg/m^2^) (24). In this study, the weight and height of participants were measured by trained staff using calibrated instruments in the physical examination. Each measurement was repeated three times, and the average value was calculated. BMI was categorized into four groups: underweight (BMI < 18.5 kg/m^2^), normal (18.5 kg/m^2^ ≤ BMI < 24.0 kg/m^2^), overweight (24.0 kg/m^2^ ≤ BMI < 28.0 kg/m^2^), and obesity (BMI ≥ 28.0 kg/m^2^) (30).

### 2.5 Assessment and classification of BP

Participants who met the eligibility criteria were instructed to rest in the seated position for at least 5 min prior to BP measurements. The trained primary care worker positioned the armband 2–3 cm above the participants' elbow joint and then ensured it was appropriately tightened. Blood pressure was measured using a standard Omron electronic sphygmomanometer, with three readings taken at 1-min intervals. Measurements were considered valid only if the difference between readings did not exceed 10 mm Hg. Finally, we took the average of the three measurements as the SBP and DBP levels. Following the 2020 global hypertension practice guidelines made by the International Society of Hypertension (6), BP is categorized into four classifications based on the SBP and DBP: normal BP (< 130 and < 85 mmHg), high-normal BP (130–139 and/or 85–89 mm Hg), grade 1 hypertension (140–159 and/or 90–99mm Hg), and grade 2 hypertension (≥160 and/or ≥100 mm Hg). Participants were further divided into controlled (< 140 and < 90 mm Hg) and uncontrolled (≥140 and/or ≥90 mm Hg) groups according to the BP levels (6).

### 2.6 Statistical analysis

Baseline characteristics of the study sample were presented as frequencies and percentages for categorical variables, and continuous variables were presented as the median and interquartile range (IQR). The Chi-square test or Kruskal–Wallis H-test was used to chart comparisons regarding variables across the different BP classifications.

We initially proposed using ordinal logistic regression to analyze the factors influencing BP classifications. However, after adjusting for confounders, the parallel lines test yielded a *P*-value of 0.028 (< 0.05), indicating that the proportional odds assumption was not met. As a result, multinomial logistic regression was used to explore the associations between lifestyle behaviors, BMI (as independent variables), and BP classifications (as dependent variables). Furthermore, multiplicative interactions were assessed in the multinomial logistic regression by creating product terms of BMI with each lifestyle behavior score to investigate the modifying effects. Stratified analyses were conducted based on the lifestyle behaviors and BMI across different BP classifications. Finally, to ensure the robustness of the results, we further grouped the participants according to BP control status, and the stratified analyses of BMI subgroups were performed in the binomial logistic regression. Of note, the effect of the interaction between lifestyle behaviors and BMI was also assessed in the case of BP control.

All statistical analyses were performed using SPSS (version 26.0, IBM), and *P* < 0.05 (two-tailed) was considered to be significant.

## 3 Results

### 3.1 Baseline characteristics of study participants

Among the 17,441 participants, 10,254 (58.79%) were female, 25.32 % had normal BP, 19.47 % had high-normal BP, 36.95 % had grade 1 hypertension, and 18.26 % had grade 2 hypertension. Additionally, the median and IQR of BMI was 24.61 (22.32–27.06) kg/m^2^, including 39.31% of normal, 2.98% of underweight, 39.59% of overweight, and 18.12% of obesity. The characteristics of participants with BP classifications are shown in Table 1. Overall, there were significant differences across BP classifications in the distribution of age, gender, education, annual household income, taking antihypertensive medicine, BMI, smoking status, drinking status, dietary patterns, and PA levels (*P* < 0.05). Compared with those with normal BP, individuals with abnormal BP (including high-normal BP, grade 1 hypertension, and grade 2 hypertension) were more likely to be female (55.07% vs. 56.33%, 59.44%, and 65.27%), have a lower educational level (75.77% vs. 77.50%, 79.47%, and 82.42%), have a lower annual household income (69.16% vs. 71.08%, 71.66%, and 71.43%), take no antihypertensive medicine (15.81% vs. 18.20%, 19.62%, and 23.01%), and be obese (15.38% vs. 17.61%, 18.84%, and 21.00%). In terms of lifestyle behaviors, participants with grade 2 hypertension were more likely to have low PA levels (15.06% vs. 17.46%), and less likely to follow a low-risk dietary pattern (4.35% vs. 3.01%) compared to those with normal blood pressure.

**Table 1:** Baseline characteristics of study participants.

**Characteristics**	**Total population (*n =* 17,441)**	**BP classifications**				***P-*value**
		**Normal BP (*****n** =* **4,416)**	**High-normal BP (*****n** =* **3,396)**	**Grade 1 hypertension (*****n** =* **6,444)**	**Grade 2 hypertension (*****n** =* **3,185)**	
**Age (years)**	73(69–77)	72(69–77)	72(69–77)	73(69–77)	73(70–78)	<0.001
**Gender**						<0.001
Male	7,187 (41.21)	1,984 (44.93)	1,483 (43.67)	2,614 (40.56)	1,106 (34.73)	
Female	10,254 (58.79)	2,432 (55.07)	1,913 (56.33)	3,830 (59.44)	2,079 (65.27)	
**Education**						<0.001
Primary school or below	13,724 (78.69)	3,346 (75.77)	2,632 (77.50)	5,121 (79.47)	2,625 (82.42)	
Middle school	2,819 (16.16)	798 (18.07)	592 (17.43)	984 (15.27)	445 (13.97)	
High school or above	898 (5.15)	272 (6.16)	172 (5.06)	339 (5.26)	115 (3.61)	
**Annual household income (yuan)**						<0.001
< 10,000	12,361 (70.87)	3,054 (69.16)	2,414 (71.08)	4,618 (71.66)	2,275 (71.43)	
10,000–19,999	2,573 (14.75)	618 (13.99)	477 (14.05)	957 (14.85)	521 (16.36)	
20,000–34,999	1,316 (7.55)	367 (8.31)	266 (7.83)	468 (7.26)	215 (6.75)	
≥35,000	1,191 (6.83)	377 (8.54)	239 (7.04)	401 (6.22)	174 (5.46)	
**Taking antihypertensive medicine**						<0.001
No	3,313 (19.00)	698 (15.81)	618 (18.20)	1,264 (19.62)	733 (23.01)	
Yes	14,128 (81.00)	3,718 (84.19)	2,778 (81.80)	5,180 (80.38)	2,452 (76.99)	
**BMI**						<0.001
Obesity	3,160 (18.12)	679 (15.38)	598 (17.61)	1,214 (18.84)	669 (21.00)	
Overweight	6,905 (39.59)	1,719 (38.93)	1,381 (40.67)	2,561 (39.74)	1,244 (39.06)	
Underweight	520 (2.98)	165 (3.74)	112 (3.30)	147 (2.28)	96 (3.01)	
Normal	6,856 (39.31)	1,853 (41.96)	1,305 (38.43)	2,522 (39.14)	1,176 (36.92)	
**Smoking status**						<0.001
Current	1,889 (10.83)	548 (12.41)	378 (11.13)	684 (10.61)	279 (8.76)	
Former	2,208 (12.66)	610 (13.81)	471 (13.87)	788 (12.23)	339 (10.64)	
Never	13,344 (76.51)	3,258 (73.78)	2,547 (75.00)	4,972 (77.16)	2,567 (80.60)	
**Drinking status**						<0.001
Current	675 (3.87)	174 (3.94)	146 (4.30)	258 (4.00)	97 (3.05)	
Former	1,022 (5.86)	317 (7.18)	216 (6.36)	362 (5.62)	127 (3.99)	
Never	15,744 (90.27)	3,925 (88.88)	3,034 (89.34)	5,824 (90.38)	2,961 (92.97)	
**Dietary patterns**						0.008
High-risk	16,733 (95.94)	4,224 (95.65)	3,243 (95.49)	6,177 (95.86)	3,089 (96.99)	
Low-risk	708 (4.06)	192 (4.35)	153 (4.51)	267 (4.14)	96 (3.01)	
**PA levels**						0.030
Low	2,774(15.91)	665(15.06)	535(15.75)	1,018(15.80)	556(17.46)	
Moderate	5,381(30.85)	1,416(32.07)	1,025(30.18)	1,946(30.20)	994(31.21)	
High	9,286(53.24)	2,335(52.88)	1,836(54.06)	3,480(54.00)	1,635(51.33)	
**Sleeping status**						0.103
High-risk	8,479 (48.62)	2,165 (49.03)	1,655 (48.73)	3,063 (47.53)	1,596 (50.11)	
Low-risk	8,962 (51.38)	2,251 (50.97)	1,741 (51.27)	3,381 (52.47)	1,589 (49.89)	

BP, blood pressure; BMI, Body Mass Index; PA, physical activity.

### 3.2 Association of lifestyle behaviors and BMI with BP classifications

**Figure 1:**
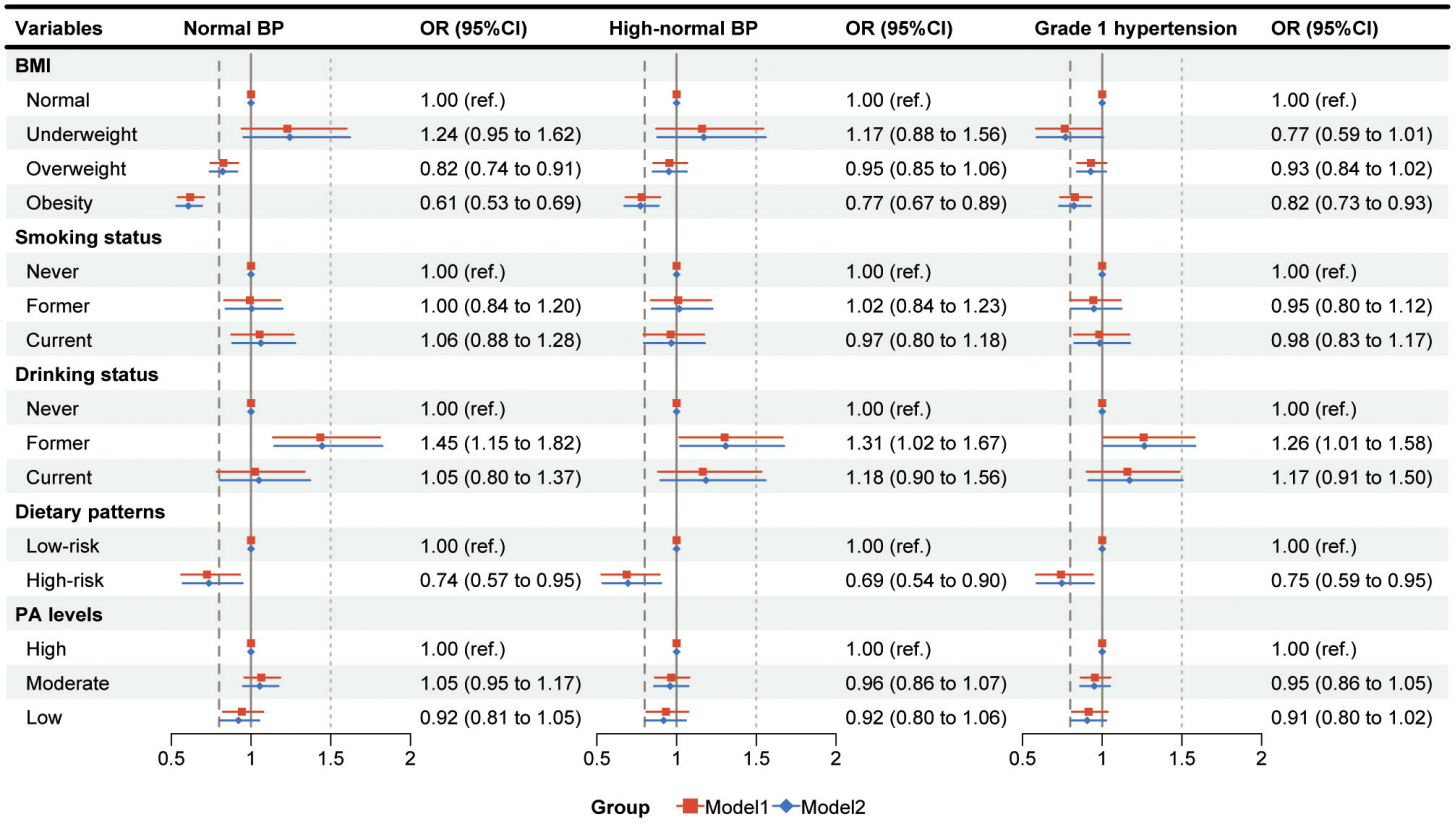
The associations between influencing factors and blood pressure classifications. Multinomial logistic regression was used to analyze the effects of lifestyle behaviors and BMI on BP classifications, with grade 2 hypertension as the reference group; Model 1, adjusted for age, gender, education, and annual household income; Model 2, adjusted for age, gender, education, annual house-hold income, and taking antihypertensive medicine; BP, blood pressure; BMI, Body Mass Index; PA, physical activity; OR, odds ratio; CI, confidence interval.

After adjusting for potential factors, the results showed that compared with Grade 2 hypertension, obesity was significantly associated with lower odds of exhibiting lower levels of BP classifications. Specifically, compared to participants with a normal BMI, those with obesity were less likely to have normal BP (OR: 0.61, 95% CI: 0.53–0.69), high-normal BP (OR: 0.77, 95% CI: 0.67–0.89), and grade 1 hypertension (OR: 0.82, 95% CI: 0.73–0.93). Similarly, compared with never drinking, the BP classifications of former drinking status had higher odds of being normal BP (OR: 1.45, 95% CI: 1.15–1.82), High-normal BP (OR: 1.31, 95% CI: 1.02–1.67), and Grade 1 hypertension (OR: 1.26, 95% CI: 1.01–1.58), respectively. Moreover, participants with a high-risk dietary pattern were less likely to have relatively lower BP classifications compared with Grade 2 hypertension such as normal BP (OR: 0.74, 95% CI: 0.57–0.95), high-normal BP (OR: 0.69, 95% CI: 0.54–0.90), and grade 1 hypertension (OR: 0.75, 95% CI: 0.59–0.95) (Figure 1, Supplementary Table 1).

### 3.3 Interaction between lifestyle behaviors and BMI on BP classifications

As shown in Supplementary Table 2, the effect of interaction expressed by product terms between lifestyle behaviors and BMI was further estimated in the multinomial logistic regression. After adjusting for potential confounders, significant multiplicative interactions between drinking status and BMI on normal BP, high-normal BP, and grade 1 hypertension were found both in Model 1 and Model 2 (all *P*_forinteraction_ < 0.001). Additionally, we also observed a borderline but non-significant interaction between dietary patterns and BMI on high-normal BP and grade 1 hypertension (*P*_forinteraction_ = 0.062 and 0.065, respectively).

### 3.4 Association of different subgroups of drinking status and BMI with BP classifications

**Figure 2:**
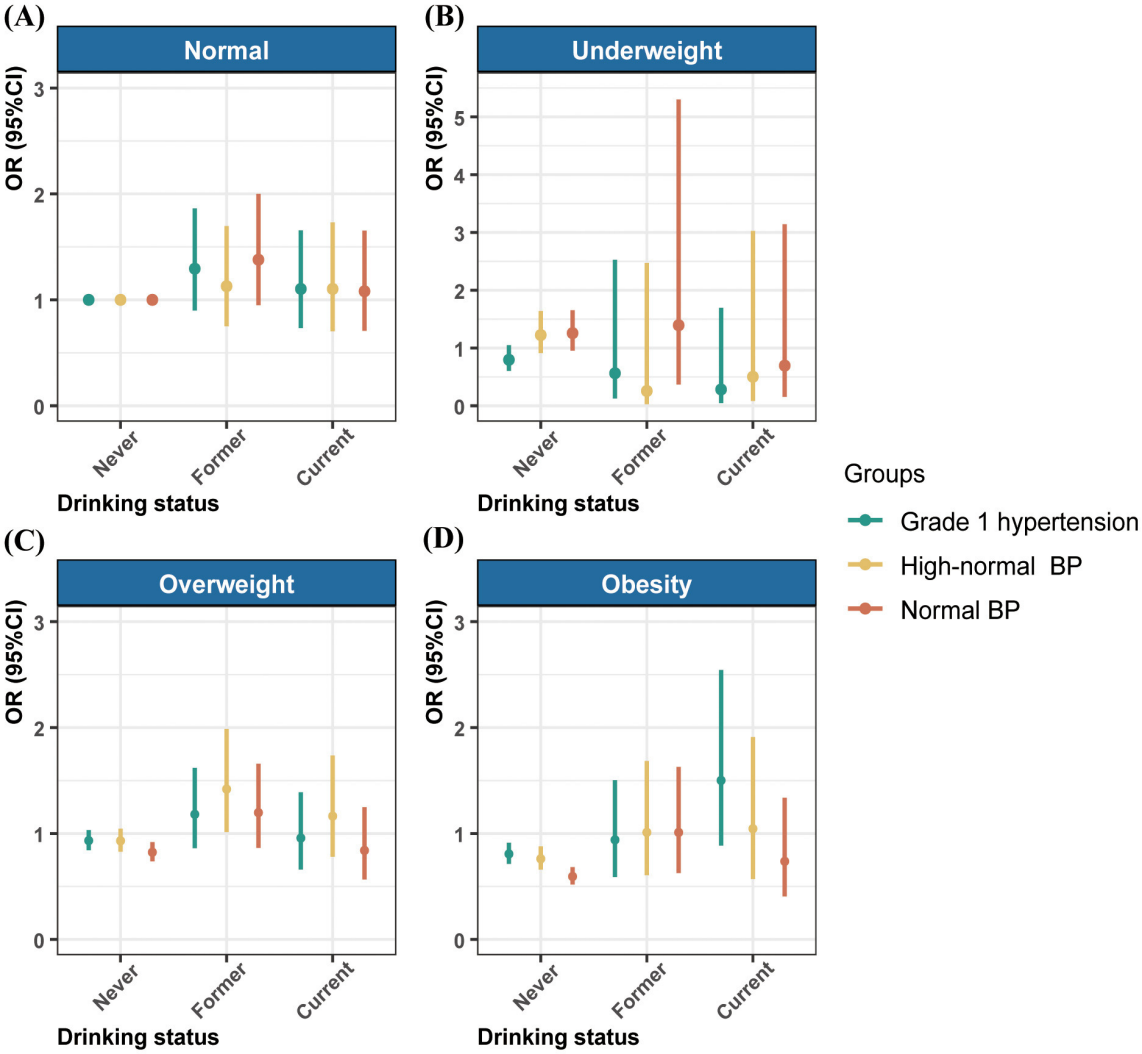
Association of drinking status with the blood pressure classifications by body mass index. The results adjusted for age, gender, education, annual household income, and taking antihypertensive medicine. BP, blood pressure; OR, odds ratio; CI, confidence interval.

The results indicated that compared to participants who never drank with normal BMI, those who also never drank but had elevated BMI were less likely to fall into lower BP classifications. For example, compared with Grade 2 hypertension, the OR (95% CI) for normal BP, high-normal BP, and grade 1 hypertension in never drinking with obese individuals were 0.59 (0.52–0.68), 0.76 (0.66–0.88), and 0.81 (0.71–0.91), respectively. Furthermore, among overweight individuals who had former drinking status, the OR (95% CI) of high-normal BP compared with grade 2 hypertension was 1.42 (1.01–1.99) (Figure 2, Supplementary Table 3).

### 3.5 Interaction between lifestyle behaviors and BMI on BP Control

**Figure 3:**
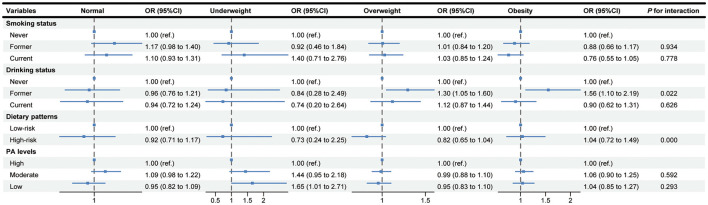
Association of lifestyle behaviors with blood pressure control stratified by body mass index subgroups. The results adjusted for age, gender, education, annual household income, and taking antihypertensive medicine. PA, physical activity; OR, odds ratio; CI, confidence interval.

The associations of lifestyle behaviors with BP control stratified by BMI subgroups are shown in Figure 3. Compared with participants who never drink, the OR (95% CI) of BP control in those with former drinking status were 1.30 (1.05–1.60) and 1.56 (1.10–2.19) among overweight and obesity groups, respectively. Similarly, in the group with underweight, the OR (95% CI) of BP control in participants with a low PA level was 1.65 (1.01–2.71) compared to the high PA level. Additionally, significant multiplicative interactions were found between BMI and former drinking, and the high-risk dietary pattern (all *P*_forinteraction_ < 0.05).

## 4 Discussion

In this cross-sectional study, we included 17,441 older adults with hypertension and observed that BMI and lifestyle behaviors such as the high-risk diet pattern, and former alcohol drinking were independently associated with the severity of BP classification, respectively. Furthermore, the current study observed a multiplicative interaction between drinking status and BMI on BP classifications. Specifically, among non-alcohol drinkers, higher BMI corresponded to the higher severity of BP classifications. Among overweight participants, those with a history of former alcohol consumption were more likely to exhibit better BP classifications. Similar results were observed in BMI-stratified analyses of BP control. Furthermore, low PA level was significantly associated with more effective BP control in underweight individuals. These findings underscore the critical role of healthy lifestyles and body weight management in controlling BP among older adults with hypertension.

The current study revealed that unhealthy dietary patterns, characterized by insufficient intake of vegetables and fruits and a high consumption of red meat, increased the severity of BP classifications in older adults with hypertension. Consistent with our findings, studies from West Africa reported that low consumption of vegetables and fruits contributed to poor BP control among Nigerian adults with hypertension (31, 32). Additionally, excessive red meat consumption was proven to further elevate BP levels in adults with risk factors for CVD (33). Conversely, diets rich in vegetables and fruits and lower in meats (excluding fish) substantially reduced BP levels and the risk of developing high BP (34, 35). According to the current literature, potassium, one of the most essential dietary components for decreasing BP levels (36), comes mainly from vegetables and fruits (37), while it is less in red meat (38). Thus, the use of potassium-rich Dietary Approaches to Stop Hypertension diet therapy (39) to control BP levels is crucial for individuals with hypertension, and the reduction is progressively greater when baseline SBP levels are high (40).

Growing evidence has indicated that alcohol intake, particularly excessive drinking, significantly raised BP levels among adults with hypertension (41, 42). A systematic review and meta-analysis demonstrated that reducing alcohol consumption within the recommended threshold range could significantly decrease BP in a dose-dependent manner both in healthy participants and hypertensive individuals (43). Interestingly, our study found that among older adults with hypertension, former drinkers had lower blood pressure severity compared to those who had never consumed alcohol. A previous study on alcohol consumption and cardiovascular health indicated that compared with non-wine drinkers, those who drink moderately or lightly tend to have better blood pressure levels (44). The reason for this phenomenon may be the “healthy drinker effect” (45), which suggests that drinkers tend to have better BP levels than non-drinkers due to other health-related factors except alcohol consumption. Similar results were also observed in the subsequent stratified analyses. Among the overweight subgroup, former drinkers had lower severity of BP classifications and better effect of BP control. The possible explanation may be that the impact of BMI on BP levels diminishes as BMI increases (46). Thus, alcohol consumption may have a more significant impact on BP than BMI. The findings could not, however, be directly compared with established evidence due to a lack of corresponding studies. More researches are still warranted to explore the potential impact of former drinking on BP.

Additionally, numerous studies have demonstrated that an increased risk of elevated BP and hypertension was related to the increasing trends in BMI. Consistent with our study, Dey et al. (47) investigated the factors influencing the control of diabetes and hypertension in South India, and the findings indicated that higher BMI corresponds to higher levels of BP among hypertensive patients. In brief, overweight and obese individuals were shown to exhibit higher BP levels and poorer BP control compared to those with normal weight. Notably, evidence suggests that prolonged sedentary behavior, as an adverse lifestyle factor, is associated with increased obesity risk in older adults (48). Concurrently, a bibliometric analysis indicated that sedentary behavior not only compromises blood pressure control efficacy but also elevates cardiovascular disease risk (49). Consequently, implementing the “sitting less and moving more” principle (50) combined with moderate weight reduction is critical for lowering blood pressure levels and cardiovascular risk in hypertensive patients with obesity (51, 52). Moreover, our study examined the combined impact of lifestyle behaviors and BMI on BP levels. We found a significant multiplicative interaction between drinking status and BMI in relation to BP classifications and control. Similar results were noted in a longitudinal study conducted in China that observed a significant multiplicative interaction between overweight/obesity and drinking/light drinking/heavy drinking among male participants (17). We found that the severity of BP classifications increased with BMI among non-alcohol drinkers, suggesting that individuals who never consume alcohol should pay more attention to managing their BMI. From a public health perspective, the findings of our study could help to identify hypertensive older adults with poorer BP control, who may benefit more from BP reduction through effective BMI management combined with lifestyle modifications.

Notably, our study found that BP control was better in low PA levels among underweight participants. The result may be attributed to a healthy metabolism with lower Triglyceride and Low-density lipoprotein cholesterol levels and higher High-density lipoprotein cholesterol levels in underweight individuals (53). Despite relatively low levels of PA, higher than predicted resting energy expenditure is a more potent driver of BP control. However, more exploration is still needed to further distinguish the healthy underweight population and those who lost weight for illness and eating disorders to examine the robustness of the results.

### 4.1 Study strengths and limitations

This study has several strengths. Firstly, it specifically targets older adults with hypertension, alleviating the critical issue of an aging population and the rising prevalence of hypertension in China. Secondly, it examines the effect of lifestyle behaviors and BMI and their potential interactions on BP classifications, highlighting the importance of encouraging older adults to adopt a healthy lifestyle and manage their BMI to reduce the severity of BP classifications. Thirdly, we further conducted the stratified analyses across various BMI subgroups to determine which kinds of participants are more likely to have optimal or more severe BP classifications.

Nevertheless, our study has certain limitations. Firstly, as with all cross-sectional studies, the causal relationship between lifestyle behaviors, BMI, and BP classifications cannot be established because all variables are measured at the same time. Secondly, the lifestyle behaviors were self-reported, which may introduce recall bias. As a result, the collected information may underestimate the true relationship between lifestyle behaviors, BMI, and BP classifications. Thirdly, although this study included most lifestyle behaviors related to BP that have been examined in previous studies, some factors such as alcohol consumption were still not assessed in detail. Further investigation is needed to explore the specific effects of alcohol consumption on BP. Fourthly, our findings, based on hypertensive patients aged 65 years or older, may not be generalizable to the broader population across all age groups. Finally, given the complexity of real-world settings and the specific characteristics of the study population, this study could not entirely rule out the influence of antihypertensive medication on BP measurements, despite efforts to minimize confounding. Future research should focus on optimizing study designs that balance real-world applicability with methodological rigor.

## 5 Conclusions

In summary, the findings in our study revealed the link between poor dietary patterns, obesity with severe BP classifications. An interaction effect between drinking status and BMI has also been observed in relation to BP classifications and control. Overweight/obesity may also contribute to elevated blood pressure levels among individuals who do not consume alcohol. Based on these findings, older adults with hypertension, particularly non-drinkers, are encouraged to adopt healthier dietary habits and actively manage their weight. Primary healthcare workers should play a proactive role in promoting lifestyle modifications to improve hypertension management in rural settings.

## Data Availability

The raw data supporting the conclusions of this article will be made available by the authors, without undue reservation.
