# Supplementary material for: Association between lifestyle behaviors and body mass index with blood pressure classifications among older adults with hypertension in China
Source: Front Public Health. 2025 Jul 3;13:1610715. doi: 10.3389/fpubh.2025.1610715 (PMC12267040; doi:10.3389/fpubh.2025.1610715)
Supplement: Supplementary file 1 [file Data_Sheet_1.pdf]

Supplementary Material

1 Supplementary Figures and Tables

1.1 Supplementary Figures

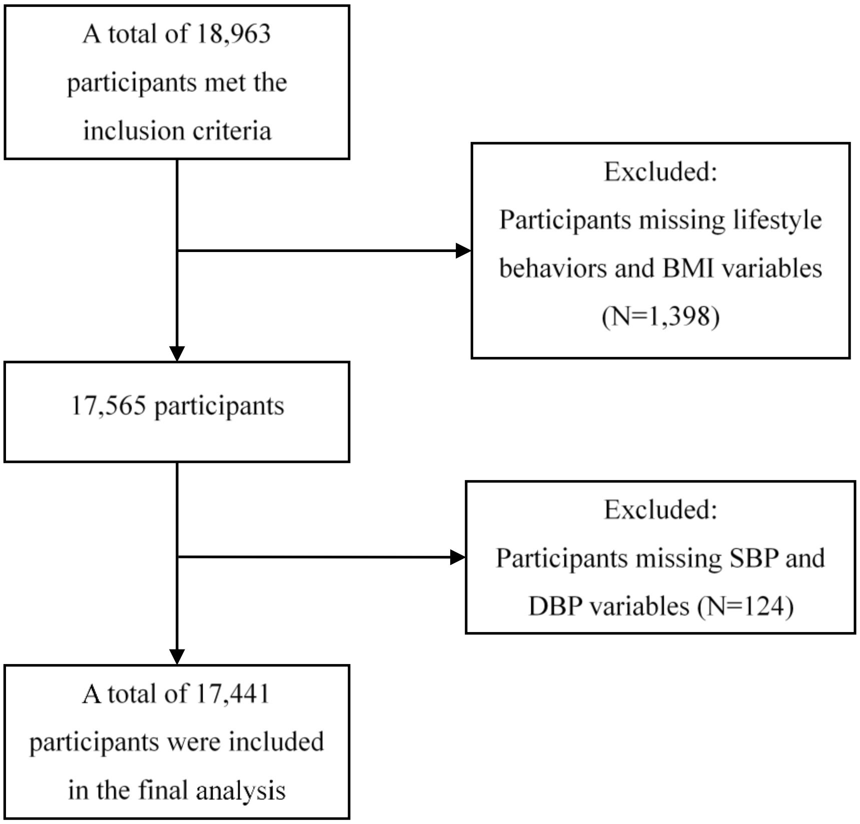

Supplementary Figure 1. Inclusion and exclusion of participants. BMI, Body Mass Index; SBP, Systolic Blood Pressure; DBP, Diastolic Blood Pressure.

1.2 Supplementary Tables

Supplementary Table 1. Multinomial logistic regression analysis for lifestyle behaviors, Body Mass Index, and blood pressure classifications.

Characteristics	Model1			Model2		
	**Normal BP OR (95%CI)**	**High to normal BP OR (95%CI)**	**Grade 1 hypertension OR (95%CI)**	**Normal BP OR (95%CI)**	**High-normal BP OR (95%CI)**	**Grade 1 hypertension OR (95%CI)**
**Age (years)**	0.97**(0.96 to 0.98)	0.98**(0.97 to 0.99)	0.98 **(0.98 to 0.99)	0.97**(0.96 to 0.98)	0.98**(0.97 to 0.99)	0.98**(0.98 to 0.99)
**Gender**						
Female	Ref.	Ref.	Ref.	Ref.	Ref.	Ref.
Male	1.37**(1.20 to 1.55)	1.36**(1.19 to 1.55)	1.24**(1.10 to 1.39)	1.34**(1.18 to 1.52)	1.34**(1.18 to 1.53)	1.23*(1.09 to 1.38)
**Education**						
High school or above	Ref.	Ref.	Ref.	Ref.	Ref.	Ref.
Middle school	0.84 (0.65 to 1.08)	0.95 (0.73 to 1.24)	0.78*(0.61 to 0.99)	0.85 (0.67 to 1.10)	0.96 (0.73 to 1.26)	0.78*(0.61 to 1.00)
Primary school or below	0.75*(0.59 to 0.94)	0.87 (0.68 to 1.12)	0.78*(0.63 to 0.98)	0.75*(0.60 to 0.95)	0.88 (0.68 to 1.13)	0.79*(0.63 to 0.99)
**Annual household income (yuan)**						
≥35,000	Ref.	Ref.	Ref.	Ref.	Ref.	Ref.
20,000 to 34,999	0.80 (0.62 to 1.02)	0.90(0.69 to 1.17)	0.96 (0.75 to 1.22)	0.80 (0.62 to 1.02)	0.90 (0.69 to 1.17)	0.96 (0.75 to 1.22)
10,000 to 19,999	0.56**(0.45 to 0.70)	0.68*(0.54 to 0.86)	0.83 (0.67 to 1.02)	0.57*(0.46 to 0.71)	0.68*(0.54 to 0.87)	0.83 (0.67 to 1.02)
<10,000	0.66**(0.54 to 0.80)	0.81*(0.66 to 1.00)	0.93 (0.77 to 1.12)	0.66**(0.54 to 0.80)	0.81 *(0.66 to 1.00)	0.93 (0.77 to 1.12)
**Taking antihypertensive medicine**						
Yes	—	—	—	Ref.	Ref.	Ref.
No	—	—	—	0.62**(0.55 to 0.70)	0.74**(0.66 to 0.84)	0.81**(0.73 to 0.90)
**BMI**						
Normal	Ref.	Ref.	Ref.	Ref.	Ref.	Ref.
Underweight	1.23 (0.94 to 1.60)	1.16 (0.87 to 1.54)	0.77 (0.59 to 1.00)	1.24 (0.95 to 1.62)	1.17 (0.88 to 1.56)	0.77 (0.59 to 1.01)

Continued Supplementary Table 1. Multinomial logistic regression analysis for lifestyle behaviors, Body Mass Index, and blood pressure classifications.

Characteristics	Model1			Model2		
	**Normal BP OR (95%CI)**	**High to normal BP OR (95%CI)**	**Grade 1 hypertension OR (95%CI)**	**Normal BP OR (95%CI)**	**High-normal BP OR (95%CI)**	**Grade 1 hypertension OR (95%CI)**
Overweight	0.83**(0.75 to 0.92)	0.96 (0.85 to 1.07)	0.93 (0.84 to 1.03)	0.82**(0.74 to 0.91)	0.95 (0.85 to 1.06)	0.93 (0.84 to 1.02)
Obesity	0.62**(0.54 to 0.71)	0.78**(0.68 to 0.90)	0.83*(0.74 to 0.93)	0.61**(0.53 to 0.69)	0.77**(0.67 to 0.89)	0.82*(0.73 to 0.93)
**Smoking status**						
Never	Ref.	Ref.	Ref.	Ref.	Ref.	Ref.
Former	0.99 (0.83 to 1.19)	1.01 (0.84 to 1.22)	0.94 (0.80 to 1.12)	1.00 (0.84 to 1.20)	1.02 (0.85 to 1.23)	0.95 (0.80 to 1.12)
Current	1.05 (0.88 to 1.27)	0.96 (0.79 to 1.17)	0.98 (0.82 to 1.17)	1.06 (0.88 to 1.28)	0.97 (0.80 to 1.18)	0.99 (0.83 to 1.17)
**Drinking status**						
Never	Ref.	Ref.	Ref.	Ref.	Ref.	Ref.
Former	1.44*(1.14 to 1.81)	1.30*(1.02 to 1.67)	1.26*(1.01 to 1.58)	1.45*(1.15 to 1.82)	1.31*(1.02 to 1.67)	1.26*(1.01 to 1.59)
Current	1.02 (0.79 to 1.34)	1.16 (0.88 to 1.53)	1.16 (0.90 to 1.49)	1.05 (0.81 to 1.37)	1.18 (0.90 to 1.56)	1.17 (0.91 to 1.50)
**Dietary patterns**						
Low-risk	Ref.	Ref.	Ref.	Ref.	Ref.	Ref.
High-risk	0.72*(0.56 to 0.93)	0.69*(0.53 to 0.89)	0.74*(0.58 to 0.94)	0.74*(0.57 to 0.95)	0.70*(0.54 to 0.90)	0.75*(0.59 to 0.95)
**PA levels**						
High	Ref.	Ref.	Ref.	Ref.	Ref.	Ref.
Moderate	1.07 (0.96 to 1.18)	0.97 (0.86 to 1.08)	0.95 (0.87 to 1.05)	1.06 (0.95 to 1.17)	0.96 (0.86 to 1.07)	0.95 (0.86 to 1.05)
Low	0.94 (0.83 to 1.08)	0.93 (0.81 to 1.07)	0.92 (0.81 to 1.03)	0.92 (0.81 to 1.05)	0.92 (0.80 to 1.06)	0.91 (0.80 to 1.02)

Take the grade 2 hypertension as the reference group. **P* value＜0.05, ***P* value＜0.001; BP, blood pressure; BMI, Body Mass Index; PA, physical activity; OR, odds ratio; CI, confidence interval; Model 1, adjusted for age, gender, education, and annual household income; Model 2, adjusted for age, gender, education, annual household income, and taking antihypertensive medicine.

Supplementary Table 2. Effect of interactions between lifestyle behaviors and Body Mass Index on blood pressure classifications.

Variables	Normal BP<br>*P* for interaction	High-normal BP<br>*P* for interaction	Grade 1 hypertension<br>*P* for interaction
**Model 1**			
BMI*Smoking status	0.197	0.984	0.907
BMI*Drinking status	<0.000	<0.000	<0.000
BMI*PA levels	0.751	0.615	0.142
BMI*Dietary patterns	0.379	0.052	0.058
BMI*Sleeping status	0.335	0.785	0.209
**Model 2**			
BMI*Smoking status	0.173	0.954	0.887
BMI*Drinking status	<0.000	<0.000	<0.000
BMI*PA levels	0.968	0.471	0.101
BMI*Dietary patterns	0.449	0.062	0.065
BMI*Sleeping status	0.269	0.715	0.239

BP, blood pressure; BMI, Body Mass Index; PA, physical activity; Model 1, adjusted for age, gender, education, and annual household income; Model 2, adjusted for age, gender, education, annual household income, and taking antihypertensive medicine.

Supplementary Table 3. Association of drinking status with the blood pressure classifications stratified by Body Mass Index.

Characteristics	Blood pressure classifications		
	**Normal BP OR (95%CI)**	**High-normal BP OR (95%CI)**	**Grade 1 hypertension OR (95%CI)**
**Age (years)**	0.97**(0.96 to 0.98)	0.98**(0.97 to 0.98)	0.98**(0.98 to 0.99)
**Gender**			
Female	Ref.	Ref.	Ref.
Male	1.36**(1.22 to 1.51)	1.33**(1.19 to 1.49)	1.20**(1.09 to 1.33)
**Education**			
High school or above	Ref.	Ref.	Ref.
Middle school	0.85 (0.66 to 1.10)	0.96 (0.73 to 1.25)	0.78*(0.61 to 1.00)
Primary school or below	0.75*(0.59 to 0.94)	0.87 (0.67 to 1.11)	0.78*(0.63 to 0.98)
**Annual household income (yuan)**			
≥35,000	Ref.	Ref.	Ref.
20,000 to 34,999	0.79 (0.62 to 1.02)	0.90 (0.69 to 1.17)	0.96 (0.75 to 1.22)
10,000 to 19,999	0.56**(0.45 to 0.70)	0.68*(0.54 to 0.86)	0.83 (0.67 to 1.02)
<10,000	0.65**(0.54 to 0.79)	0.80*(0.65 to 0.98)	0.92 (0.76 to 1.11)
**Taking antihypertensive medicine**			
Yes	Ref.	Ref.	Ref.
No	0.62**(0.55 to 0.70)	0.74**(0.66 to 0.83)	0.82**(0.74 to 0.90)
**BMI*Drinking status**			
Normal*Never	Ref.	Ref.	Ref.
Normal*Former	1.38 (0.95 to 2.00)	1.13 (0.75 to 1.70)	1.29 (0.90 to 1.86)
Normal*Current	1.08 (0.71 to 1.65)	1.10 (0.70 to 1.73)	1.10 (0.73 to 1.66)
Underweight*Never	1.26 (0.96 to 1.65)	1.22 (0.91 to 1.64)	0.80 (0.60 to 1.05)
Underweight*Former	1.39 (0.37 to 5.30)	0.26 (0.03 to 2.47)	0.56 (0.13 to 2.53)
Underweight*Current	0.70 (0.15 to 3.14)	0.50 (0.08 to 3.03)	0.28 (0.05 to 1.70)
Overweight*Never	0.82**(0.74 to 0.92)	0.93 (0.83 to 1.05)	0.93 (0.84 to 1.03)
Overweight*Former	1.20 (0.86 to 1.66)	1.42*(1.01 to 1.99)	1.18 (0.86 to 1.62)
Overweight*Current	0.84 (0.57 to 1.25)	1.16 (0.78 to 1.74)	0.96 (0.66 to 1.39)
Obesity*Never	0.59**(0.52 to 0.68)	0.76**(0.66 to 0.88)	0.81*(0.71 to 0.91)
Obesity*Former	1.01 (0.63 to 1.63)	1.01 (0.61 to 1.69)	0.94 (0.59 to 1.50)
Obesity*Current	0.74 (0.41 to 1.34)	1.05 (0.57 to 1.91)	1.50 (0.89 to 2.54)

*P value$<$0.05, **P value$<$0.001; BP, blood pressure; BMI, Body Mass Index; OR, odds ratio; CI, confidence interval. The model adjusted for age, gender, education, annual household income, and taking antihypertensive medicine.
